# A pangolin-origin SARS-CoV-2-related coronavirus: infectivity, pathogenicity, and cross-protection by preexisting immunity

**DOI:** 10.1038/s41421-023-00557-9

**Published:** 2023-06-17

**Authors:** Xing-Yao Huang, Qi Chen, Meng-Xu Sun, Hang-Yu Zhou, Qing Ye, Wu Chen, Jin-Yu Peng, Yi-Ni Qi, Jun-Qiong Zhai, Ying Tian, Zi-Xin Liu, Yi-Jiao Huang, Yong-Qiang Deng, Xiao-Feng Li, Aiping Wu, Xiao Yang, Guan Yang, Yongyi Shen, Cheng-Feng Qin

**Affiliations:** 1 State Key Laboratory of Pathogen and Biosecurity, Beijing Institute of Microbiology and Epidemiology, AMMS, Beijing, China; 2 Institute of Systems Medicine, Chinese Academy of Medical Sciences & Peking Union Medical College, Beijing, China; 3 Guangzhou Zoo & Guangzhou Wildlife Research Center, Guangzhou, Guangdong China; 4 State Key Laboratory for Animal Disease Control and Prevention, Guangdong Laboratory for Lingnan Modern Agriculture, Center for Emerging and Zoonotic Diseases, College of Veterinary Medicine, South China Agricultural University, Guangzhou, Guangdong China; 5 State Key Laboratory of Proteomics, Beijing Proteome Research Center, National Center for Protein Sciences (Beijing), Beijing Institute of Lifeomics, Beijing, China; 6 Guangdong Provincial Key Laboratory of Zoonosis Prevention and Control, Guangzhou, Guangdong China; 7 Research Unit of Discovery and Tracing of Natural Focus Diseases, Chinese Academy of Medical Sciences, Beijing, China

**Keywords:** Mechanisms of disease, Cell biology

## Abstract

Virus spillover remains a major challenge to public health. A panel of SARS-CoV-2-related coronaviruses have been identified in pangolins, while the infectivity and pathogenicity of these pangolin-origin coronaviruses (pCoV) in humans remain largely unknown. Herein, we comprehensively characterized the infectivity and pathogenicity of a recent pCoV isolate (pCoV-GD01) in human cells and human tracheal epithelium organoids and established animal models in comparison with SARS-CoV-2. pCoV-GD01 showed similar infectivity to SARS-CoV-2 in human cells and organoids. Remarkably, intranasal inoculation of pCoV-GD01 caused severe lung pathological damage in hACE2 mice and could transmit among cocaged hamsters. Interestingly, in vitro neutralization assays and animal heterologous challenge experiments demonstrated that preexisting immunity induced by SARS-CoV-2 infection or vaccination was sufficient to provide at least partial cross-protection against pCoV-GD01 challenge. Our results provide direct evidence supporting pCoV-GD01 as a potential human pathogen and highlight the potential spillover risk.

## Introduction

The ongoing coronavirus disease 2019 (COVID-19) pandemic caused by severe acute respiratory syndrome coronavirus 2 (SARS-CoV-2) and its variants continues to thrive worldwide, posing unprecedented challenges to public health and socioeconomic development. The origin and evolution of SARS-CoV-2 remain some of the most controversial and mysterious issues in the scientific community and the public.

Human coronaviruses mostly originate from animal hosts, and bats are considered to be the natural reservoir hosts of many coronaviruses^1–3^. Numerous SARS-like bat coronaviruses (bCoV) have been identified with high genetic similarity (genomic identity > 90%) with SARS-CoV-2. Among these bCoVs, RaTG13 detected from the samples of Yunnan Rhinolophus was considered to have the closest relationship with SARS-CoV-2 until several BANAL bCoVs were found in Laos at the beginning of 2022^4,5^. Although the newly identified BANAL bCoVs were shown to infect and replicate in some human cells, most bCoVs were not isolated or showed limited replication in human cells^5,6^. Structural and biochemical analyses also supported the weak ACE2 binding affinity of the bCoV RBD and limited infectivity to human cells^7^.

The discovery and identification of SARS-CoV-2-related coronaviruses in pangolins were somewhat unexpected. Pangolins are the only scaled mammal and the most trafficked mammal that is mainly distributed in Asia and Africa^8,9^. A number of viral agents have been identified in pangolins^10,11^, and the global illicit trade of pangolins increases the risks of spillover of pangolin viruses to humans. During the search for the natural host of SARS-CoV-2, we and another group independently identified SARS-CoV-2-related pangolin coronaviruses (pCoVs) in trafficked Malayan pangolins^12,13^. Compared with bCoVs, some pCoVs showed high binding affinity to ACE2, the receptor of SARS-CoV-2^14^, and functional assays with pseudovirus confirmed that pCoVs are capable of entering a panel of human cells^2,15^. Importantly, bioinformatic analysis suggested that the entire receptor binding domain (RBD) of SARS-CoV-2 was introduced through recombination with pCoVs, representing a critical step in the evolution of SARS-CoV-2 to acquire the capability to infect humans^16,17^. However, human infections with pCoVs have not yet been documented, and the potential risk of spillover of pCoVs to humans remains incompletely investigated.

Herein, we comprehensively characterize the infectivity and pathogenicity of a representative pCoV in comparison with SARS-CoV-2 in cells, organoids, and animal models. Meanwhile, the cross-protection of host immunity induced by SARS-CoV-2 infection or vaccination against potential pCoV infection was investigated. Our results clearly demonstrated that pCoV-GD01 may represent a potential human pathogen similar to SARS-CoV-2, and mass vaccination may have decreased the possibility of human infection.

## Results

### The pCoV-GD01 virus is highly infective to human cells and airway epithelium organoids

Phylogenetic analysis classified SARS-CoV-2-related pCoVs into two lineages: GD and GX (Supplementary Fig. S1a). Clearly, the GD lineage viruses pCoV-GD01 and GD-MP-789 have a closer genetic distance with SARS-CoV-2 than those of the GX lineage. Pairwise amino acid sequence identity analysis (Supplementary Fig. S1b) showed that the GD, GX lineage, and SARS-CoV-2 shared the same amino acid identity in nsp7, nsp12-1, and E protein, and the GD lineage had a higher amino acid identity to SARS-CoV-2 than the GX lineage in the other proteins except for the S protein. Furthermore, focusing on specific functional domains of the S protein, the GD lineage and GX lineage share the same amino acid identity with SARS-CoV-2 in the fusion peptide, heptad repeat 1, heptad repeat 2, transmembrane domain, and cytoplasmic tail. The NTD of the GX lineage exhibits higher similarity, while the GD lineage has higher similarity in the RBD region compared to SARS-CoV-2 (Supplementary Fig. S1c). Multi-sequence alignment showed that the GD lineage contained only 7 amino acid substitutions (R346T, A372T, I402V, K417R, Q498H, H519K, K529Q) in the RBD region (Supplementary Fig. S1d) compared with the SARS-CoV-2 reference strain Wuhan-Hu-1. Interestingly, some of these substitutions, including R346, K417, and Q498 mutants, have already been reported to be key for viral fitness in many Variant of Concerns (VOCs) and even animal-adapted SARS-CoV-2 strains^18^. In the present study, we chose pCoV-GD01, the closest pCoV to SARS-CoV-2, for subsequent experiments.

**Fig. 1:**
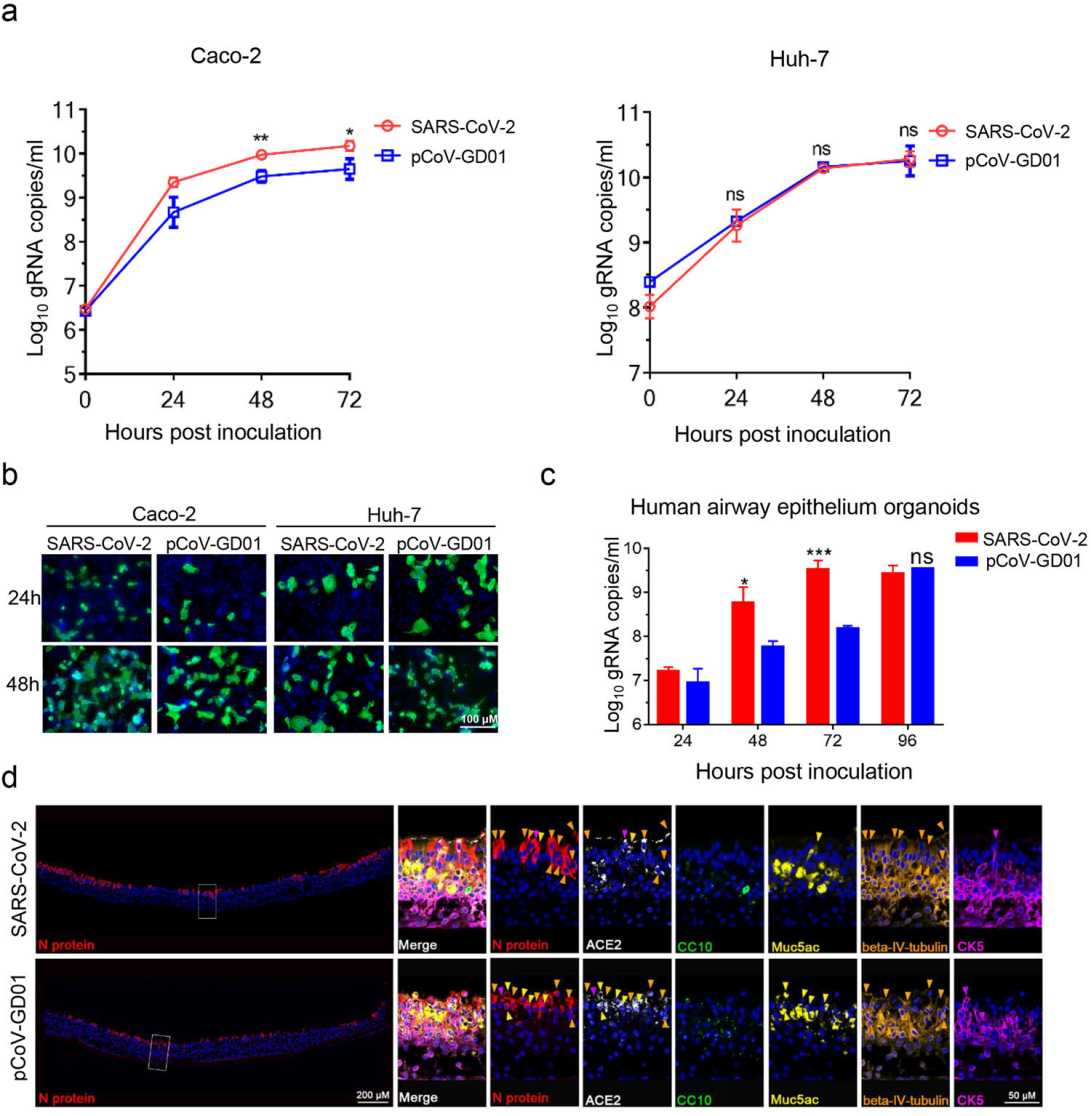
Characterization of pCoV-GD01 in human cells and epithelium airway organoids. **a** Growth curves of SARS-CoV-2 or pCoV-GD01 in Caco-2 or Huh-7 cells. Cells were inoculated with the indicated viruses at an MOI of 0.1, and the cell supernatants were collected at the indicated times for the determination of virus titers by qRT‒PCR. The data are presented as means ± SD. Student’s *t*-test was performed for statistical analysis (*P < 0.05; **P < 0.01; ns, not significant). **b** IF staining of Caco-2 or Huh-7 cells inoculated with SARS-CoV-2 or pCoV-GD01 at an MOI of 0.1 at 24 and 48 hpi with SARS-CoV-2 N protein antibodies. **c** Human airway epithelium organoids were inoculated with SARS-CoV-2 or pCoV-GD01, and viral gRNA in the topical secretions was detected at the indicated times using qRT‒PCR to determine the virus titers. The assay was performed in three replicates. The data are presented as means ± SD. Student’s *t*-test was performed for statistical analysis (*P < 0.05; ***P < 0.001; ns, not significant). **d** Multiplex IF staining analysis for the inoculated cells in human airway epithelium organoids. hACE2 (white), Clara cells stained with CC10 (green), goblet cells stained with Muc5ac (yellow), ciliated cells with beta-IV-tubulin (gold), basal cells with CK5 (magenta). The white frame was magnified on the right. The green arrows indicate CoV^+^/CC10^+^ cells, the yellow arrows indicate CoV^+^/Muc5ac^+^ cells, the gold arrows indicate CoV^+^/beta-IV-tubulin^+^ cells, and the magenta arrows indicate CoV^+/^CK5^+^ cells.

To characterize the in vitro phenotypes, the infection and growth properties of pCoV-GD01 and SARS-CoV-2 were determined in several SARS-CoV-2-susceptible cell lines, including Vero, Caco-2, and Huh-7 cells. Both pCoV-GD01 and SARS-CoV-2 caused obvious cytopathic effects (CPE) (Supplementary Fig. S2a) and formed visible plaques (Supplementary Fig. S2b) in Vero cells. Immunofluorescence staining (IF staining) and qRT‒PCR assays also showed that pCoV-GD01 is fully capable of replicating and peaked at similar levels in Vero cells (Supplementary Fig. S2c, d). Similarly, pCoV-GD01 infection in Caco-2 and Huh-7 cells led to an obvious CPE at 96 h post-inoculation (hpi) (Supplementary Fig. S3). Meanwhile, both pCoV-GD01 and SARS-CoV-2 were amplified efficiently and peaked at 72 hpi in Caco-2 and Huh-7 cells (Fig. 1a). Viral protein expression in pCoV-GD01- and SARS-CoV-2-infected Caco-2 and Huh-7 cells was also comparable by IF staining (Fig. 1b).

We further characterized the infectivity and replication kinetics of pCoV-GD01 in the well-established human airway epithelium organoid model^19,20^. Multiplex IF staining assays showed that the human airway epithelium organoids used in our study were differentiated into a structured layer consisting of ciliated cells, goblet cells, basal cells, and Clara cells (Supplementary Fig. S4a, b). Upon SARS-CoV-2 infection, viral RNA in the topical secretion increased rapidly and peaked at 72 hpi. Similarly, the viral RNA load of pCoV-GD01 peaked at 96 hpi, with a delayed increasing trend compared with SARS-CoV-2 (Fig. 1c). Furthermore, multiplex IF staining showed that pCoV-GD01 predominantly infected goblet cells (49.4%) and ciliated cells (24.5%) in human airway epithelium organoids (Fig. 1d; Supplementary Fig. S5a, b), which was similar to SARS-CoV-2.

As SARS-CoV-2 and pCoV-GD01 infection did not cause obvious disorganization of human airway epithelium organoids, we further sought to determine the host response to pCoV-GD01 infection. A Luminex cytokine assay showed that 19 of 34 tested cytokines were upregulated in response to pCoV-GD01 infection. In particular, IL-4, IL-9, IL-15, and TNF-beta were the most abundant cytokines (Supplementary Fig. S6). Meanwhile, 8 cytokines, including IL-6, TNF-alpha, IL-1alpha, IL-1RA, IL-7, IP-10, MCP-1, and RANTES, were downregulated in response to pCoV-GD01. More importantly, the cytokine heatmap showed similar trends in response to either SARS-CoV-2 or pCoV-GD01 infection.

Transcription profiling of human airway epithelium organoids infected with pCoV-GD01 or SARS-CoV-2 showed quite consistent results (Supplementary Fig. S7a). Both pCoV-GD01 and SARS-CoV-2 infection readily stimulated the transcription of a large panel of genes related to viral infection or antiviral response, including IFIT1, IFIT2, IFIT3, STAT2, OSA3, IP-10, etc. Gene Ontology (GO) gene set analysis also showed that both pCoV-GD01 and SARS-CoV-2 infection induced a quite similar tendency of overexpression or downregulation of genes (Supplementary Fig. S7b). Accordingly, the top-ranked pathways induced by pCoV-GD01 and SARS-CoV-2 were enriched as seen by KEGG analysis (Supplementary Fig. S8a), and the top 2 ranked signaling pathways were ribosome and coronavirus disease-COVID-19. Moreover, consistent with the results of GO gene analysis, almost all of the significantly differentially expressed genes of SARS-CoV-2 and pCoV-GD01 infection in the COVID-19 pathway had the same level of change (Supplementary Fig. S8b). These results demonstrate that similar antiviral and cytokine responses were induced in human airway epithelial cells by pCoV-GD01 and SARS-CoV-2.

### pCoV-GD01 efficiently infects hACE2 mice and causes pulmonary damage

**Fig. 2:**
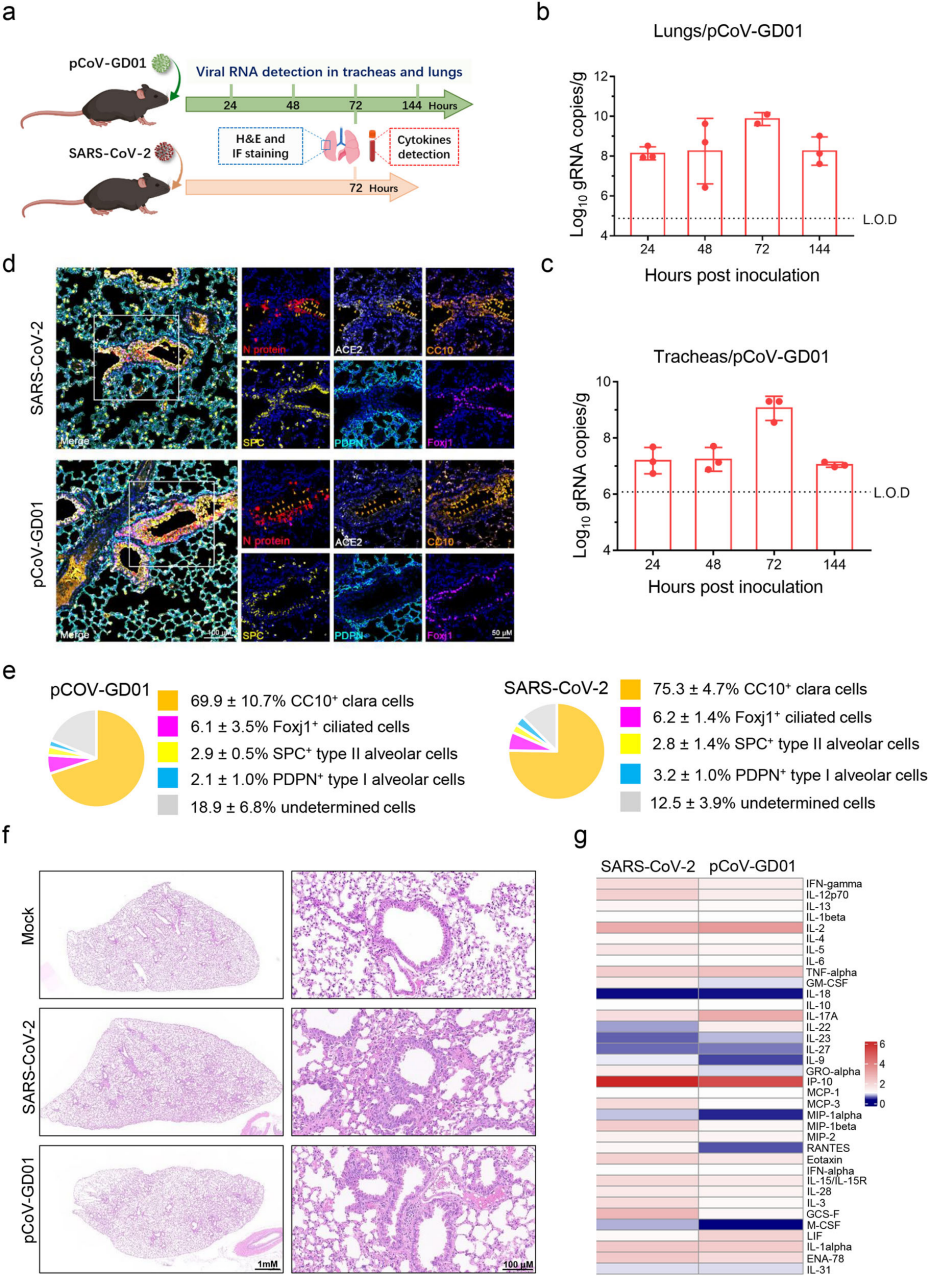
Intranasal inoculation with pCoV-GD01 in hACE2 mice. **a** 8-week-old hACE2 mice were inoculated with 6 × 10^3^ PFU of pCoV-GD01 or SARS-CoV-2 through i.n. administration, pCoV-GD01-inoculated mice were sacrificed at 24, 48, 72, and 144 h, and SARS-CoV-2-inoculated mice were sacrificed at 72 h. **b**, **c** Viral gRNA in lungs and tracheas was determined by qRT‒PCR. Data are shown as means ± SD. L.O.D, limit of detection. **d** Multiplex IF staining analysis for the inoculated cells in the lungs (left panel). hACE2 (white), Clara cells stained with CC10 (gold), alveolar type 2 (AT2) cells with SPC (yellow), alveolar type 1 (AT1) cells with PDPN (blue), and ciliated cells with Foxj (magenta). The white frame was magnified on the right. The gold arrows indicate CoV^+^ /CC10^+^ cells, the yellow arrows indicate CoV^+^/SPC^+^ cells, the blue arrows indicate CoV^+^/PDPN^+^ cells, and the magenta arrows indicate CoV^+^/Foxj1^+^ cells. **e** Percentage of each cell type with the SARS-CoV-2^+^ or pCoV-GD01^+^ population (right panel). Statistical analysis of the percentage of each cell compartment within SARS-CoV-2^+^ or pCoV-GD01^+^ cells. The data are presented as means ± SD (*n* = 3). **f** Gross necropsy and H&E staining of lung tissue sections from mice inoculated with pCoV-GD01, SARS-CoV-2, or PBS (mock) at 72 hpi. **g** Cytokine production in sera of pCoV-GD01- and SARS-CoV-2-inoculated mice. The color represents the fold change in detected cytokine concentration vs. that of MOCK samples, and the color represents the fold change in detected cytokine concentration vs. that of MOCK samples.

The humanized ACE2 (hACE2) mouse has been well developed for studying the pathogenicity and pathogenesis of SARS-CoV-2^21^. To characterize the pathogenicity of pCoV-GD01 in hACE2 mice, groups of 8-week-old hACE2 mice were subjected to intranasal (i.n.) challenge with 6 × 10^3^ PFU of pCoV-GD01 (Fig. 2a). Upon pCoV-GD01 infection, robust viral genomic RNA (gRNA) replication was readily detected in the lung and trachea samples, and the peak gRNA level reached ~10^10^ and ~10^9^ copies/g in the lungs (Fig. 2b) and tracheas (Fig. 2c), respectively, at 72 hpi. The viral sgRNA levels in the lungs and tracheas of pCoV-GD01- or SARS-CoV-2-inoculated mice were also comparable at 72 hpi (Supplementary Fig. S9). Furthermore, the pCoV-GD01 N protein predominantly colocalized with CC10^+^ Clara cells (69.9%) along the airway in the lungs (Fig. 2d, e), which was similar to SARS-CoV-2. Most importantly, hematoxylin and eosin (H&E) staining of lung sections from pCoV-GD01-infected animals showed mild interstitial pneumonia characterized by alveolar septal thickening and distinctive vascular system injury (Fig. 2f). As expected, the same dose of SARS-CoV-2 inoculum also caused similar pulmonary damage in the lungs (Fig. 2f). Meanwhile, both SARS-CoV-2 and pCoV-GD01 infection resulted in direct apoptosis (Cleaved-Casp3^+^) in the lungs of hACE2 mice (Supplementary Fig. S10). More importantly, both SARS-CoV-2 and pCoV-GD01 infection induced the production of cytokines such as IFN-gamma, IL-12p70, IL-13, IL-2, IL-5, TNF-alpha, IL-17A, IP-10, MCP-3, MIP-1beta, MIP-2, Exotaxin, IL-15/15R, IL-28, IL-3, G-CSF, IL-1alpha and ENA-78 (Fig. 2g). These results demonstrated that pCoV-GD01 could efficiently infect hACE2 mice and cause pulmonary damage.

### pCoV-GD01 could efficiently transmit among hamsters

**Fig. 3:**
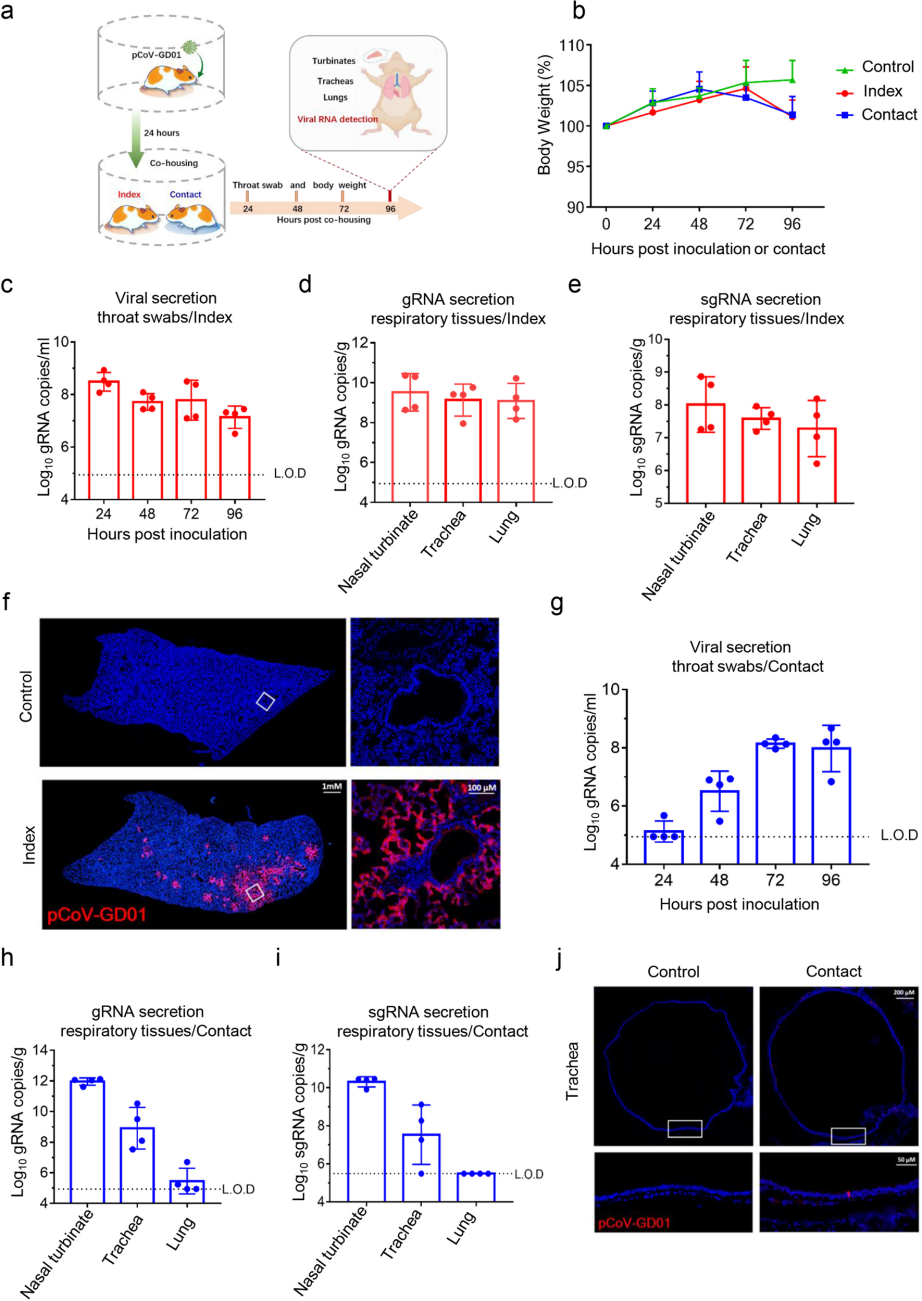
Direct contact transmission of pCoV-GD01 in hamsters. **a** Each 8- to 10-week-old male hamster that received i.n. inoculation of pCoV-GD01 was placed in an isolated cage, and then the other naïve male animal was cocaged at 24 hpi. **b** Changes in body weight of hamsters (*n* = 4). Data are shown as means ± SD. **c** Throat swabs were collected from the index (*n* = 4) animals and subjected to viral gRNA analysis. Data are shown as means ± SD. L.O.D, the limit of detection. **d**, **e** Viral gRNA and sgRNA in the lungs and tracheas of the index animals were determined by qRT‒PCR at 96 hpi. Data are shown as means ± SD. **f** IF staining analysis for index hamster lung paraffin sections for pCoV-GD01 (red) at 96 hpi. The white frame was magnified on the right. **g** Throat swabs were collected from the contact (*n* = 4) animals and subjected to viral gRNA analysis. Data are shown as means ± SD. **h**, **i** Viral gRNA and sgRNA loads in the lungs and tracheas of the contact animals were determined by qRT‒PCR at 96 hpc. Data are shown as means ± SD. **j** IF staining analysis of paraffin sections of contact hamster tracheas for pCoV-GD01 (red) at 96 hpc. The white frame is magnified below.

Syrian golden hamsters have been widely used to assess the transmissibility of SARS-CoV-2^22,23^. Here, we sought to investigate whether pCoV-GD01 could establish direct contact transmission in hamsters similar to SARS-CoV-2. Four hamsters were inoculated intranasally with pCoV-GD01 (2 × 10^4^ PFU/per hamster) and raised in an individual cage. After 24 hpi, each inoculated animal (index) was transferred to a new cage and cocaged with one naïve hamster (contact) (Fig. 3a). Animals without any treatment were set as controls. Although no obvious clinical signs of diseases (hunched posture, ruffled fur, inactivity, etc.) were observed in all animals, both index and contact animals showed weight loss at 96 hpi or contact compared with the control animals (Fig. 3b). High levels of viral gRNA secretion were detected in throat swabs of all the index animals at all test time points (Fig. 3c). Moreover, as shown in Fig. 3d, e, robust viral gRNA and sgRNA were detected in nasal turbinates, tracheas, and lungs of all index hamsters at 96 hpi. In addition, a large amount of viral N protein expression was detected in the lungs of index hamsters (Fig. 3f). These results indicated that pCoV-GD01 could effectively infect hamsters.

Intriguingly, all contact hamsters (4/4, 100%) showed increased viral gRNA secretion in throat swabs 48 hours post-contact (hpc) (Fig. 3g). Importantly, all contact hamsters maintained high levels of gRNA and sgRNA in nasal turbinate and tracheas at 96 hpc (Fig. 3h, i), while no sgRNA was detected in all lungs from contact animals (Fig. 3i). Furthermore, pCoV-GD01 viral protein signals were also detected in the tracheas of the contact hamsters at 96 hpc (Fig. 3j). Taken together, our results demonstrate that pCoV-GD01 was fully capable of establishing direct contact transmission among hamsters.

### Primary SARS-CoV-2 infection protects against secondary pCoV-GD01 infection

**Fig. 4:**
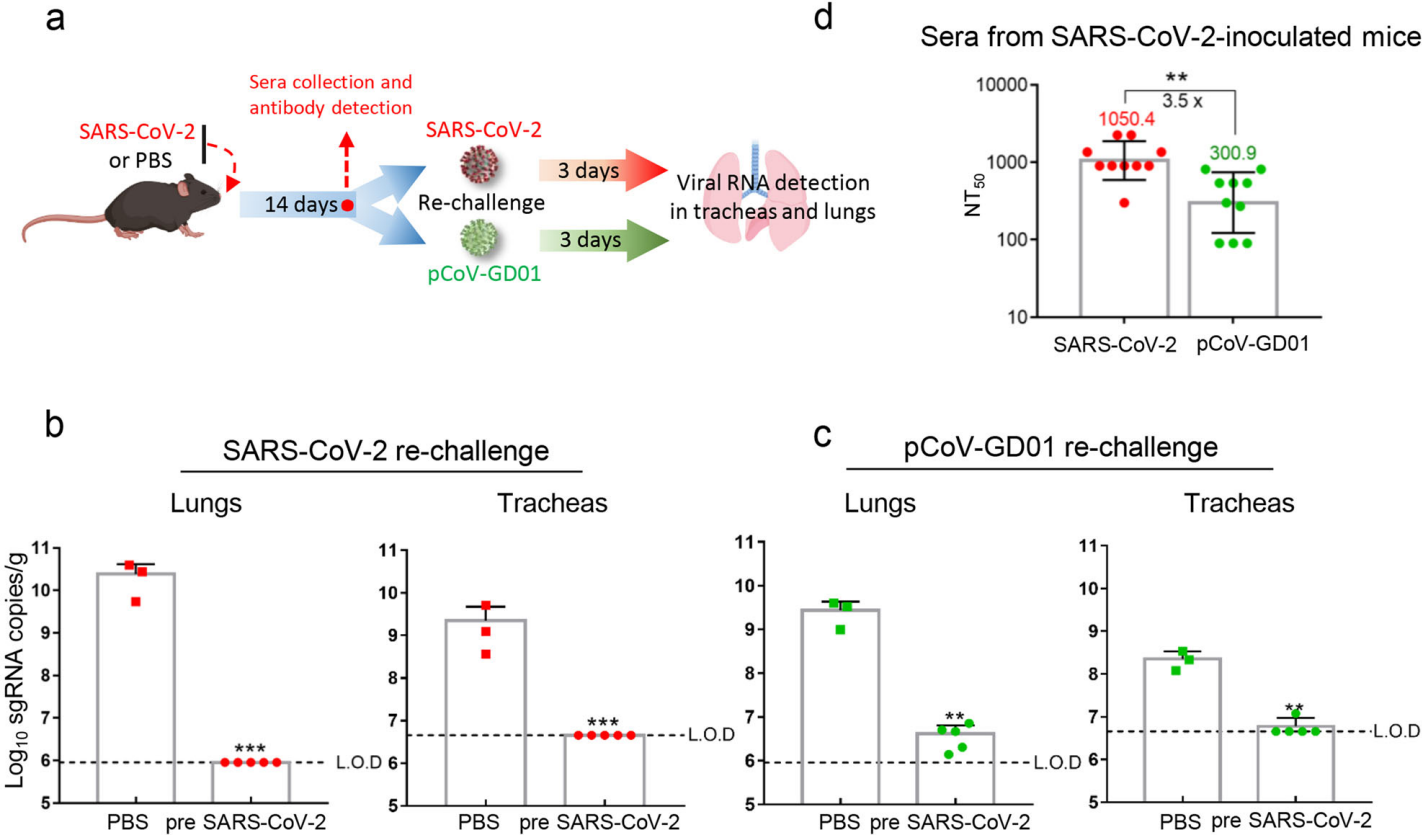
Preexisting immunity against SARS-CoV-2 provides cross-protection against pCoV-GD01. **a** 8-week-old hACE2 mice were i.n. inoculated with 6 × 10^3^ PFU SARS-CoV-2 (*n* = 10) or PBS (*n* = 6) and 14 days later inoculated with an equivalent dose of SARS-CoV-2 or pCoV-GD01. **b**, **c** Lungs and tracheas were collected 3 days post re-challenge. Viral sgRNA loads in the lungs and tracheas were determined by qRT‒PCR. Data are shown as means ± SD. **d** Sera were collected at 14 days post-inoculation and subjected to neutralization assays. The NT_50_ of sera from were analyzed using pseudovirus for SARS-CoV-2 and pCoV-GD01, respectively. Data are shown as geometric means ± SD. Student’s *t*-test was performed for statistical analysis (**P < 0.01; ***P < 0.001). L.O.D, limit of detection.

Considering the high identity of the RBD between SARS-CoV-2 and pCoV-GD01, we further sought to determine whether preexisting immunity to SARS-CoV-2 could provide cross-protection against pCoV-GD01. Groups of 8-week-old hACE2 mice were infected with SARS-CoV-2 (pre-SARS-CoV-2) or PBS. After 14 days of preinfection, each group was re-challenged with either SARS-CoV-2 or pCoV-GD01 (Fig. 4a). As shown in Fig. 4, the PBS group was highly susceptible to either SARS-CoV-2 or pCoV-GD01 challenge, and high levels of viral sgRNA were detected at 3 days post re-challenge. Remarkably, all animals preinfected with SARS-CoV-2 were protected against SARS-CoV-2 and pCoV-GD01, and only minimal viral sgRNAs were detected in the lungs and tracheas (Fig. 4b, c). We further determined the serum neutralization capability in the pre-SARS-CoV-2 group by a pseudovirus-based neutralization assay^24^. The geometric mean of the 50% neutralization titer (NT_50_) against SARS-CoV-2 and pCoV-GD01 was 1050.4 and 300.9, respectively (Fig. 4d), which indicates that SARS-CoV-2 preinfected mouse sera could cross-neutralize pCoV-GD01. As a control for homologous protection, groups of 8-week-old hACE2 mice were preinfected with pCoV-GD01 (pre pCoV-GD01) or PBS. After 14 days, the mice were re-challenged with pCoV-GD01 (Supplementary Fig. S11a). The geometric mean of the NT_50_ of the sera from the pre pCoV-GD01 group against pCoV-GD01 was 2240.7 (Supplementary Fig. S11b). As expected, the sgRNA of pCoV-GD01 in the lungs and tracheas was not detected in the pre pCoV-GD01 group (Supplementary Fig. S11c, d). Thus, prior exposure to SARS-CoV-2 confers significant cross-protection against pCoV-GD01 challenge in mice.

### SARS-CoV-2 antibodies neutralize pCoV-GD01

**Fig. 5:**
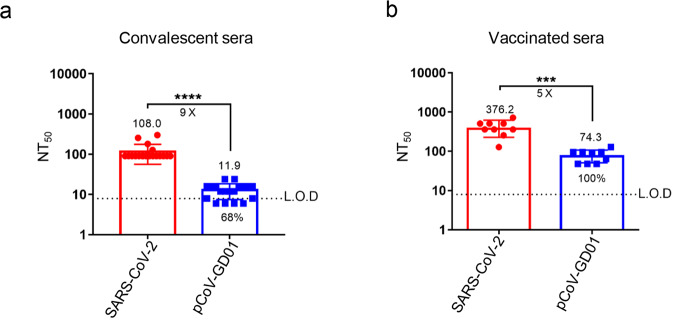
SARS-CoV-2 antibodies neutralize pCoV-GD01. **a**, **b** Convalescent sera (**a**) and ARCoV-vaccinated sera (**b**) were employed to investigate the cross-reactivity between SARS-CoV-2 and pCoV-GD01. Data are shown as geometric means ± SD. Student’s *t*-test was performed for statistical analysis (***P < 0.001; ****P < 0.0001). L.O.D, limit of detection.

Next, we sought to investigate whether the preexisting antibodies against SARS-CoV-2 were attributed to the observed protection against pCoV-GD01. A panel of human sera from COVID-19 convalescent subjects and vaccinees were subjected to a neutralization assay. We first examined the neutralization ability of sera from COVID-19 convalescent subjects (*n* = 19). Although the NT_50_ against pCoV-GD01 showed a 9.0-fold reduction in comparison with SARS-CoV-2, the majority of convalescent sera (13/19, 68%) possessed cross-neutralization activity against pCoV-GD01 (Fig. 5a). Further assays with ARCoV-vaccinated sera (*n* = 9)^25^ showed that the NT_50_ against pCoV-GD01 was 5.0-fold lower than that against SARS-CoV-2. However, all sera from vaccinees (9/9, 100%) retained neutralization activity against pCoV-GD01, and the NT_50_ value was calculated to be 74.3 (Fig. 5b). Taken together, our results indicated that neutralizing antibodies induced by SARS-CoV-2 infection or vaccination provide important cross-protection against pCoV-GD01 infection.

## Discussion

In the past twenty years, three zoonotic CoVs, SARS-CoV-2, SARS-CoV, and MERS-CoV, have spread from animals to humans and caused severe epidemic or endemic diseases^26–28^. It is estimated that ~66,000 people are silently infected with previously unknown zoonotic CoVs annually^29^. This sounds the alarm for the threat of cross-species novel emerging coronavirus diseases and raises great attention to assessing the interspecies transmission ability of these animal CoVs. The pCoV-GD01 strain used in our study has a very high genetic identity (96.7%) of the RBD with that of SARS-CoV-2^12^. Therefore, we characterized the infectivity, pathogenicity, and immune protection of pCoV-GD01 with several available experimental systems of SARS-CoV-2. Remarkably, pCoV-GD01 was fully capable of infecting human cells and airway epithelium organoids and had similar replication kinetics and CPE to SARS-CoV-2. Furthermore, the virus efficiently infects hACE2 mice and causes obvious pulmonary damage along with elevated cytokine production, including IL-2, IL-4, IL-10, IL-12p70, IL-13, IL-17A, GM-CSF, and IFN-gamma. Elevated levels of these cytokines have already been reported to be associated with severe lung injury and adverse outcomes of SARS-CoV-2 infection^30,31^.

Importantly, our study provided experimental evidence that pCoV-GD01 infection could be efficiently established via i.n. route in hamsters. More recently, another pCoV strain, pCoV-GX, was also shown to be able to be transmitted among hamsters by direct contact^32^. Moreover, a recent study demonstrated that equine ACE2 can broadly recognize SARS-CoV, SARS-CoV-2, and pCoV RBDs with high affinity^14^, and structural analysis has also revealed that pCoVs were able to utilize human ACE2 and a number of animal ACE2 orthologs for cell entry^33^. All these results highlight the potential risk of spillover from pangolins and follow-up circulation in other wild animals.

Interestingly, sera from COVID-19 patients, vaccines, and infected mice were efficient in neutralizing pCoV-GD01 (Figs. 4, 5), although the neutralization capability was reduced in comparison with SARS-CoV-2. The reduced magnitude of neutralization against pCoV-GD01 is consistent with that observed for SARS-CoV-2 variants that harbor some specific mutations in the RBD. For example, R346T in the RBD has already been reported to be associated with a milder reduction in neutralization sensitivity^34^. More importantly, prior infection with SARS-CoV-2 could partially cross-protect hACE2 mice from pCoV-GD01 challenge in vivo. These results clearly demonstrated that the preexisting immunity acquired from SARS-CoV-2 infection or vaccination could provide effective cross-protection against pCoV-GD01; thus, the risk of pCoV-GD01 spillover was also reduced during COVID-19 vaccination and SARS-CoV-2 natural infection. However, the neutralization capability as well as the potential protection against other pCoVs induced by SARS-CoV-2 infection or vaccines remains to be determined.

Overall, our results provide direct evidence that the pCoV-GD01 virus represents a potential human pathogen and that SARS-CoV-2-naïve populations might be highly susceptible to pCoV-GD01 infection. SARS-CoV-2 neutralization antibodies might have contributed to the prevention of cross-species transmission of pCoV-GD01 to humans. The accidental spillover from animals to humans deserves specific concern and extensive surveillance. Meanwhile, it is estimated that thousands of cross-species infections were caused by unknown CoVs derived from animals^29^, and further investigation of the infectivity, pathogenicity, and transmissibility of these neglected CoVs should be warranted in the future.

## Materials and methods

### Ethics statement

All animal experiments were performed in strict accordance with the guidelines set by the Chinese Regulations of Laboratory Animals and Laboratory Animal-Requirements of Environment and Housing Facilities. All animal procedures were reviewed and approved by the Animal Experiment Committee of Laboratory Animal Center, Academy of Military Medical Sciences (AMMS), China (Assurance Number: IACUC-IME-2021-014). The use of human sera has been approved in previous studies^24^.

### Cells and virus

The African green monkey kidney cell Vero (ATCC, CCL-81), Caco-2(ATCC, HTB-37), and human hepatocarcinoma cell Huh-7 (JCRB, 0403) were maintained at 37 °C under 5% CO_2_ in Dulbecco’s modified Eagle’s medium (DMEM) supplemented with 10% fetal bovine serum (FBS, Gibco), 10 mM HEPES, and 1% penicillin-streptomycin. The SARS-CoV-2 strain BetaCoV/Bei-jing/IMEBJ05/2020 (No. GWHACBB01000000) was originally isolated from a COVID-19 patient. The SARS-CoV-2-related coronavirus pCoV-GD01 was originated isolated from pangolin samples obtained by anti-smuggling operations in the Guangdong province of China^12^. For virus propagation, Vero cells were incubated with SARS-CoV-2 or pCoV-GD01, and the culture supernatants were collected at 3 days post-inoculation. The stock of SARS-CoV-2 or pCoV-GD01 was serially diluted and titrated on monolayers of Vero cells. Studies with infectious SARS-CoV-2 and pCoV-GD01 were conducted under biosafety level 3 (BSL3) facilities at the Beijing Institute of Microbiology and Epidemiology, AMMS.

### In vitro replication

Growth curves of pCoV-GD01 and SARS-CoV-2 in Vero, Huh-7, and Caco-2 cells were performed in a 12-well plate. Cells were inoculated with pCoV-GD01 or SARS-CoV-2 at a multiplicity of infection (MOI) of 0.1 for 1 h. The inoculum was removed and washed three times with PBS to remove unbound viruses, before adding fresh DMEM, supplemented with 2% FBS. Cell supernatants were collected at successive 24 h intervals post-inoculation. Viral RNA was extracted from the culture supernatant after each passage using the QIAamp Viral RNA Kit (Qiagen) and detected using qRT‒PCR. SARS-CoV-2 and pCoV-GD01 RNA were measured with the following primer-probe shown in Supplementary Table S1. Amplification was performed using a One Step PrimeScript RT-PCR Kit (Takara Bio, Otsu, Japan), and the following qRT‒PCR conditions were applied: 42 °C for 5 min and 95 °C for 10 s followed by 40 cycles of 95 °C for 5 s and 60 °C for 20 s. The PCR was conducted in a LightCycler® 480 Instrument (Roche Diagnostics Ltd). The absolute quantification of SARS-CoV-2 or pCoV-GD01 RNA levels were performed by comparison to a standard curve and is shown as RNA copies/mL.

### Infectivity assay of pCoV-GD01 and SARS-CoV-2 in human airway epithelium organoids

Human airway epithelium organoids (gifted from Prof. Li-Liang Lab). Human airway epithelium organoids were generated in an air-liquid interface for 4–6 weeks to form well-differentiated, polarized cultures that resemble in vivo pseudostratified mucociliary epithelium. Before inoculation, the apical surface of well-differentiated human airway epithelium organoid cells was washed three times with PBS. 50 μL of pCoV-GD01 or SARS-CoV-2 inoculum with a titer of 1.5 × 10^5^ PFU was added to a single organoid containing well of a round-bottom 24-well plate and incubated at 37 °C for 2 h. The inoculum was removed and washed three times with PBS. Each virus-infected organoid was then transferred to a single well of a 24-well plate (Corning) containing 500 μL of serum-free growth medium (Stem cell Technologies) and incubated at 37 °C with 5% CO_2_. At 24, 48, 72, 96 hpi, 30 μL of PBS was applied to the apical surface of cell cultures and collected after incubation for 10 min at 37 °C. Viral RNA copies were quantified by qRT‒PCR and cytokine secretion assay as described below. SARS-CoV-2- or pCoV-GD01-infected organoids were collected and subjected to multiplex IF staining as described below. Total RNAs from the SARS-CoV-2 or pCoV-GD01-infected organoids were extracted by Trizol (Invitrogen) at indicated times and subjected to RNA sequencing.

### pCoV-GD01 or SARS-CoV-2 infection in hACE2 mice

The animal experiment procedure was reviewed and approved by the Laboratory Animal Center, AMMS. hACE2 mice used in this study were generated by inserting the hACE2 gene into exon2, the first coding exon, of mouse ACE2 located in chromosome X GRC m38.p6. of zygotes of C57BL/6 mice as we reported previously^21^. For i.n. infection, 6 × 10^3^ PFU of SARS-CoV-2 or pCoV-GD01 was instilled into the nasal cavity of 8-week-old hACE2 mice anesthetized with sodium pentobarbital at a dose of 50 mg/kg by the intraperitoneal route. Mice were monitored and euthanized at indicated times to isolate tissues. The lungs and tracheas of hACE2 mice were collected for detection of viral loads using qRT‒PCR. The absolute quantification of SARS-CoV-2 or pCoV-GD01 RNA levels of indicated tissues were performed by comparison to a standard curve and is shown as RNA copies/g.

### pCoV-GD01 contact transmission in hamsters

For direct contact transmission experiments, four 8- to 10-week-old hamsters that were i.n. challenged with the pCoV-GD01 (2 × 10^4^ PFU/ per hamster), were caged for 24 h. Each pCoV-GD01-challenged hamster (index) was then transferred to a new cage containing one naïve hamster (contact). Viral RNA in throat swabs of naive hamsters were detected using qRT‒PCR at 24 to 96 h post-direct contact. The indicated tissues of index and contact hamsters were collected 96 hpi and 96 hpc for detection of viral loads using qRT‒PCR.

### IF staining of infected cells

Cells were fixed with 4% (w/v) paraformaldehyde in PBS at room temperature (RT) for 15 min. Then cells were blocked in PBS buffer containing 10% donkey serum and 0.3% Triton X-100 (Sigma) for 1 h at RT, followed by incubation with the primary antibodies CoV N protein (Sino-biological, 1:2000) at 4 °C overnight with 5% donkey serum and 0.15% Triton X-100. Cryosections were incubated with secondary antibodies diluted in 5% donkey serum and 0.15% Triton X-100 for 1 h. Nuclei were counterstained with Hoechst 33342 DNA dye (CST, 1:1000) at RT for 10 min and mounted on glass slides. Images were taken using a Carl Zeiss LSM710 confocal microscope.

### Multiplex IF staining

The 3-μm-thick paraffin sections were deparaffinized in xylene and rehydrated in a series of graded alcohols. Antigen retrieval was performed in citrate buffer (pH 6) by heating in a microwave (Sharp) for 20 min at 95 °C followed by a 20 min cool-down period at RT. Multiplex fluorescence labeling was performed using TSA-dendron-fluorophores (NEON 9-color Allround Discovery Kit for FFPE, Histova Biotechnology). Briefly, endogenous peroxidase was quenched in 3% H_2_O_2_ for 20 min, followed by treatment with blocking reagent for 30 min at RT. The primary antibody was incubated for 2–4 h in a humidified chamber at 37 °C, followed by detection using the HRP-conjugated secondary antibody and TSA-dendronfluorophores. Then, the primary and secondary antibodies were thoroughly eliminated by heating the slides in retrieval/elution buffer (Histova Biotechnology) for 10 s at 95 °C using a microwave. In a serial fashion, each antigen was labeled with distinct fluorophores. The multiplex antibody panels applied in this study were as follows: ACE2 (Abcam, 1:200); CoV N protein (Sinobiological, 1:2000); CC10 (Millipore, 1:500); FOXJ1(Abcam, 1:1000); β-IV-tubulin IV (Abcam, 1:1000); Podoplanin (Sino biological, 1:1000); SPC (Abcam, 1:500); CK5 (Abcam, 1:500); Muc5ac (ThermoFisher, 1:200); Cleaved caspase-3 (CST, 1:300); After all the antibodies were detected sequentially, the slides were imaged using the confocal laser scanning microscopy platform Zeiss LSM880.

### Deep sequencing analysis

Viral RNA was used to synthesize the first-strand cDNA by reverse transcription using SuperScript VILO cDNA Synthesis Kit (Thermo Fisher) according to the manufacturer’s protocol. The sequencing library was constructed using Ion Ampliseq Library Kit 2.0 (Thermo Fisher), and sequenced on an Ion Torrent S5Plus sequencer (Thermo Fisher). The sequencing data were mapped to the reference genome of SARS-CoV-2 (NC_045512.2) using CLC Genomic Workbench 20 (Qiagen).

### RNA library construction and sequencing

The SARS-CoV-2- or pCoV-GD01-infected organoids as previously described were used for RNA-seq. Total RNA from organoids were extracted using Trizol and DNaseI (NEB), respectively. Sequencing libraries were generated using NEBNext® UltraTM RNA Library Prep Kit for Illumina® (NEB) following the manufacturer’s recommendations and index codes were added to attribute sequences to each sample. Clustering of the index-coded samples was performed on a cBot cluster generation system using a HiSeq PE Cluster Kit v4-cBot-HS (Illumina) according to the manufacturer’s instructions. After cluster generation, the libraries were sequenced on the Illumina NovaSeq 6000 platform, and 150-bp paired-end reads were generated. After sequencing, a Perl script was used to filter the original data (raw data) to clean reads by removing contaminated reads for adapters and low-quality reads. Clean reads were aligned to the Human genome build 38 (hg38) using Hisat2 v2.1.0. The number of reads mapped to each gene in each sample was counted by HTSeq v0.6.0.

### Phylogenetic tree

Sequences of SARS-CoV-2, SARS-CoV-2 related pCoVs and bCoVs were aligned with MAFFT^35^ v7.450. The sequence information of CoVs was provided in Supplementary Table S2. Phylogenetic trees of full-length and RBD nucleotide sequences were constructed using iqtree^36^ v2.1.3 with 1,000 bootstrap replicates, employing General Time Reversible (GTR) nucleotide substitution model plus four discrete free rate categories. Whilst, a phylogenetic tree of RBD amino acid sequences was constructed with 1000 bootstrap replicates, employing WAG model^37^ plus two discrete free rate categories. All the phylogenetic trees were visualized by ggtree^38^ v3.0.4.

### Sequence identity

The sequence identity of pairwise amino acid sequences was calculated by sitePath^39^ v1.9.2.1 and visualization by pheatmap^40^ v1.0.12. The whole genome sequence similarity was calculated by Simplot^41^ v3.5.1 and visualized by ggplot2^42^ v3.3.5. The screening window size was set as 1000 bp and the step size was set as 100 bp.

### Differential expression genes

DESeq2^43^ v1.32.0 was used for differential gene expression analysis. Genes with Padj < 0.05, |Log_2_FoldChange| > 1 and mean base count > 100 were identified as differentially expressed genes (DEGs). The DEGs and identified were used as queries to search for enriched biological processes (Gene Ontology BP) and KEGG pathway using clusterProfiler^44^ v4.0.5. Heatmaps, volcano plots were constructed using pheatmap^40^ v1.0.12, EnhancedVolcano^45^ v1.10.0, while dotplot and barplot were constructed by ggplot2^42^ v3.3.5.

### H&E staining

For histopathology, lung tissues from hACE2 mice were fixed in 4% neutral-buffered formaldehyde for 72 h, embedded in paraffin, sectioned, and stained with hematoxylin and eosin. Images were captured using Olympus BX51 microscope equipped with a DP72 camera. Original magnification was 25×.

### Cytokine secretion assay

A total of 25 μL in vivo or in vitro samples was adopted for cytokine analysis with Mouse Cytokine & Chemokine Panel 1A (36 plex) (Invitrogen) according to the manufacturer’s instructions. The data were collected on Luminex 200 and analyzed by Luminex PONENT (Thermo Fisher).

### Convalescent sera and vaccine sera

The sera of 19 convalescent patients from the Fifth Medical Center of Chinese PLA General Hospital in China, Beijing (Supplementary Table S3). All participants signed an informed consent form before enrollment, and the study was reviewed and approved by the Fifth Medical Center of Chinese PLA General Hospital (2020031D). Another cohort of sera from nine participants in the phase 1 clinical trial of ARCoV was tested for neutralization against SARS-CoV-2 and pCoV-GD01. All samples were collected on day 43 post-initial immunization with 15 μg of ARCoV^25^. The sera were processed under the protocol and informed consent was approved by the Clinical Trial Ethics Committee of Shulan (Hangzhou) Hospital (YW2020-031-01). This study was conducted in accordance with the principles of Declaration of Helsinki and Good Clinical Practice.

### Microneutralization assay

In order to test the cross-neutralization activity of COVID-19 patient convalescent serum or vaccination serum against pCoV-GD01, a CPE-based micro-neutralization assay was performed as previously described. Briefly, serum samples were heat-inactivated for 30 min at 56 °C and serially diluted twofold from 1:10 to 1:1280 using DMEM (Thermo Fisher Scientific) supplemented with 2% FBS. The serum dilutions (50 μL) were then mixed with the same volume of virus solution containing 100 median tissue culture infectious dose (TCID_50_) of SARS-CoV-2 or pCoV-GD01. After incubating at 37 °C for 1 h, the serum-virus mixture was added to 96-well plates containing semiconfluent Vero cells (> 80% density). After culturing at 37 °C for 3 days, the CPE was assessed under an inverted microscope. The neutralization titer was calculated as the reciprocal of the highest sample dilution that protected 50% of the wells from CPE with the method of Reed and Muench.

### Pseudovirus-based neutralization assay

Pseudovirus-based neutralization assay. The pseudovirus-based neutralization assay of SARS-CoV-2 and pCoV-GD01 was performed as described previously^46^. Briefly, three-fold serially diluted serum was incubated with 1.3 × 10^4^ TCID_50_ SARS-CoV-2 (Beijing Yunling Biotechnology Co., Ltd) or pCoV-GD01 (Vazyme Biotech Co., Ltd) pseudovirus at 37 °C for 1 h, respectively. Then, the mixtures were used to infect Huh-7 cells or Vero cells in 96-well plates at 37 °C for 24 h. After incubation, 150 µL of supernatant was removed, and equal volumes of luciferase reagent were added to each well and incubated for 2 min. Luciferase luminescence was measured using GloMax 96 Microplate Luminometer (Promega). The 50% NT_50_ was defined as the serum dilution at which the relative light units were reduced by 50% compared with the virus control wells.

### Quantification and statistical analysis

All data were analyzed with GraphPad Prism 8.0 software. No statistical methods were used to predetermine sample size unless indicated. The investigators were not blinded to allocation during experiments and outcome assessment unless indicated (qRT‒PCR). Unless specified, data are presented as means ± SD. Analysis of Student’s *t*-test was used to determine statistical significance among different groups (*P < 0.05; **P < 0.01; ***P < 0.001; ****P < 0.0001; ns, not significant).

## Supplementary information

Supplemental Fig S1

Supplemental Fig S2

Supplemental Fig S3

Supplemental Fig S4

Supplemental Fig S5

Supplemental Fig S6

Supplemental Fig S7

Supplemental Fig S8

Supplemental Fig S9

Supplemental Fig S10

Supplemental Fig S11

Supplemental Table S1

Supplemental Table S2

Supplemental Table S3

## Data Availability

The RNA-Seq data generated in this study have been deposited in the NCBI Gene Expression Omnibus (GEO) database under accession code GSE217796.

## Materials availability

All requests for resources and reagents should be directed to C.-F.Q. (qincf@bmi.ac.cn) and will be fulfilled after completion of a materials transfer agreement.
