# Supplementary material for: A pangolin-origin SARS-CoV-2-related coronavirus: infectivity, pathogenicity, and cross-protection by preexisting immunity
Source: Cell Discov. 2023 Jun 17;9:59. doi: 10.1038/s41421-023-00557-9 (PMC10276878; doi:10.1038/s41421-023-00557-9)
Supplement: Supplementary file 1 — Supplemental Fig S1 [file 41421_2023_557_MOESM1_ESM.pdf]

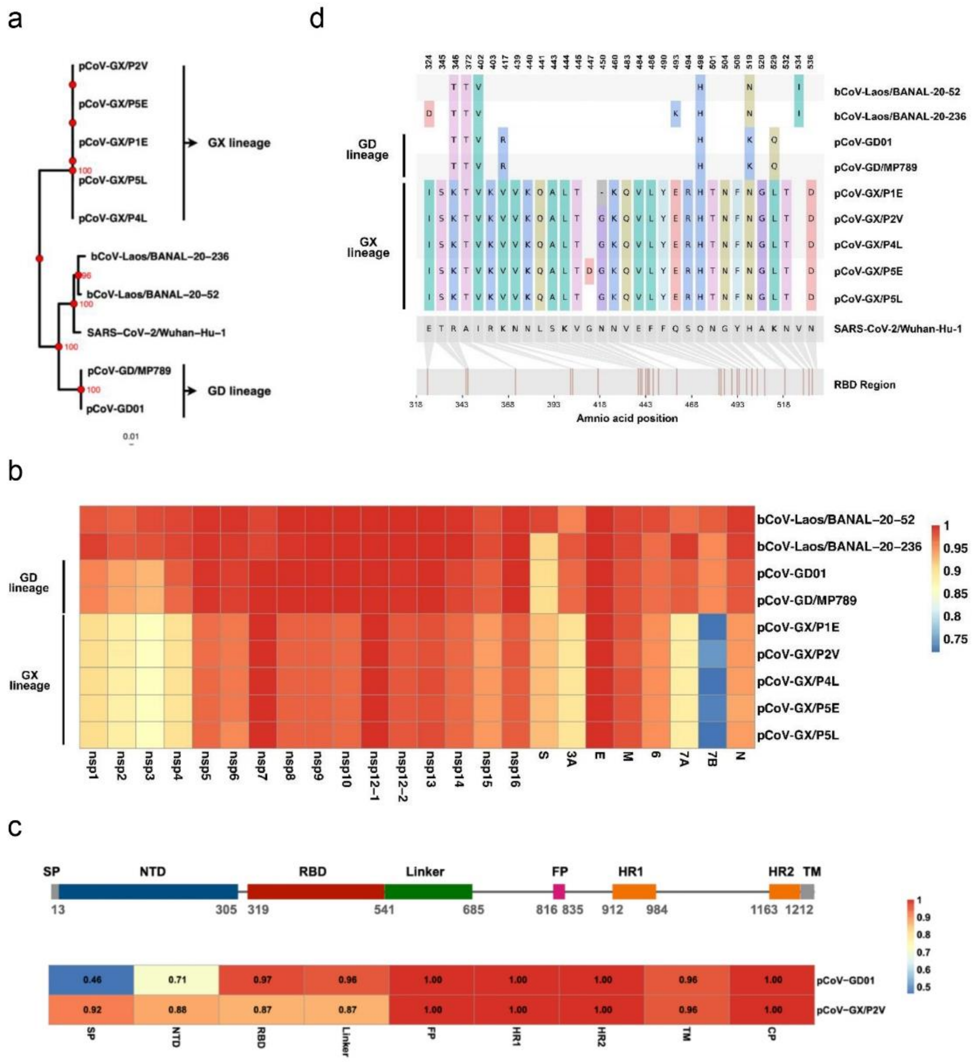

Supplementary Fig. S1 Phylogenetic analysis of SARS-CoV-2 related bCoVs and pCoVs. a Phylogenetic tree of full length nucleotide sequence of bCoVs and pCoVs related to SARS-CoV-2. The tree were build with iqtree v2.1.3 with 1,000 bootstrap replicates. **b** Pairwise amino acid sequence identities among pCoV-GD, pCoV-GX and bCoV-Laos compared to SARS-CoV-2. **c** Specific functional domains of S protein (upper pannel). Pairwise amino acid sequence identities between pCoV-GD01 and pCoV-GX/P2V compared to SARS-CoV-2 in different functional domains of S protein (bottom pannel). SP (signal peptide), NTD (N-terminal domain), RBD (receptor-binding domain), Linker (Linker of S1 and S2), FP (fusion peptide), HR1 (heptad repeat 1), HR2 (heptad repeat 2), TM (transmembrane domain), CP (cytoplasmic tail). **d** Genomic comparison of pCoV-GD, pCoV-GX and bCoV-Laos with SARS-CoV-2 in RBD region.
