# Supplementary material for: A pangolin-origin SARS-CoV-2-related coronavirus: infectivity, pathogenicity, and cross-protection by preexisting immunity
Source: Cell Discov. 2023 Jun 17;9:59. doi: 10.1038/s41421-023-00557-9 (PMC10276878; doi:10.1038/s41421-023-00557-9)
Supplement: Supplementary file 2 — Supplemental Fig S2 [file 41421_2023_557_MOESM2_ESM.pdf]

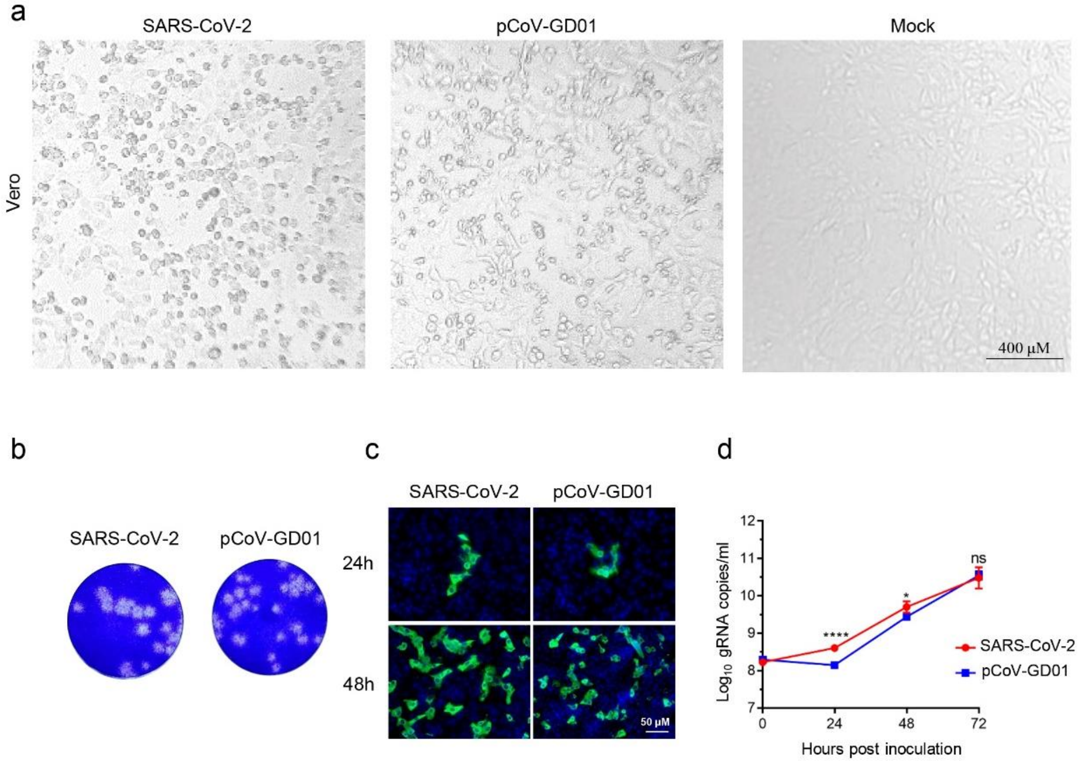

Supplementary Fig. S2 Characterization of pCoV-GD01 in Vero cells. a Microscopy image of the CPE of the virus in Vero cells. The CPE was seen in viral culture at 96 hpi. The experiment was performed twice independently in two laboratories and produced similar results. **b** Plaque morphology of SARS-CoV-2 or pCoV-GD01 on Vero cells at an MOI of 0.1. Vero cells were inoculated with the indicated viruses, and plaques were developed after 72 hours. **c** IF staining of Vero cells inoculated with SARS-CoV-2 or pCoV-GD01 at an MOI of 0.1 at 24 and 48 hpi with N protein antibodies. **d** Growth curves of SARS-CoV-2 or pCoV-GD01 in Vero cells. Vero cells were inoculated with the indicated viruses at an MOI of 0.1 and the cell supernatants were collected at the indicated times for determination of virus titers by qRT–PCR. The data are representative of at least 3 independent experiments and error bars indicate the SD. Student's t-test was performed for statistical analysis (*$p<0.05$; ****$p < 0.0001$; ns, not significant).
