# Supplementary material for: A pangolin-origin SARS-CoV-2-related coronavirus: infectivity, pathogenicity, and cross-protection by preexisting immunity
Source: Cell Discov. 2023 Jun 17;9:59. doi: 10.1038/s41421-023-00557-9 (PMC10276878; doi:10.1038/s41421-023-00557-9)
Supplement: Supplementary file 3 — Supplemental Fig S3 [file 41421_2023_557_MOESM3_ESM.pdf]

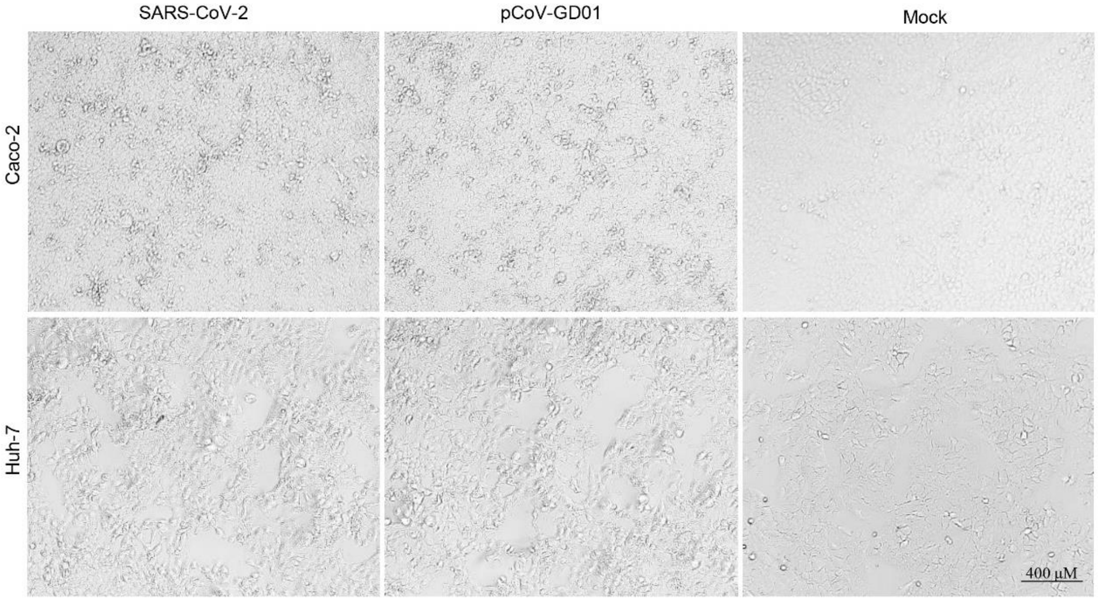

Supplementary Fig. S3 Cytopathic effect of the virus in Caco-2 and Huh-7 cells. Microscopy image of the CPE of the virus in Caco-2 or Huh-7 cells. The CPE was seen in viral culture at 96 hpi. The experiment was performed twice independently in two laboratories and produced similar results.
