# Supplementary material for: A pangolin-origin SARS-CoV-2-related coronavirus: infectivity, pathogenicity, and cross-protection by preexisting immunity
Source: Cell Discov. 2023 Jun 17;9:59. doi: 10.1038/s41421-023-00557-9 (PMC10276878; doi:10.1038/s41421-023-00557-9)
Supplement: Supplementary file 4 — Supplemental Fig S4 [file 41421_2023_557_MOESM4_ESM.pdf]

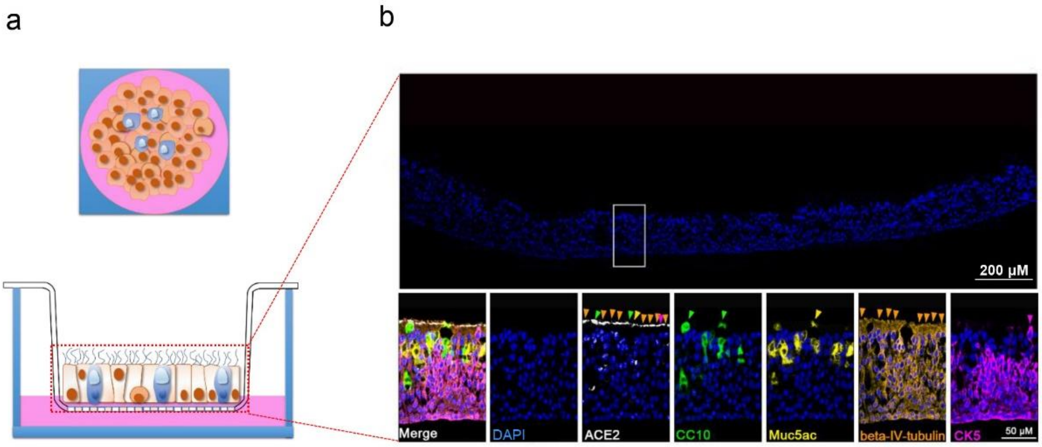

Supplementary Fig. S4 Characterization of human bronchial epithelia air-liquid interface culture. a Schematic representation of human airway epithelium organoids. White frame was magnified on the right. **b** Multiplex IF staining analysis for the hACE2-expressed cell types in of human airway epithelium organoids. Multiplex IF staining of differentiated human airway epithelium organoids revealing hACE2 (white), Clara cells with CC10 (green), goblet cells stained with Muc5ac (yellow), ciliated cells with beta-IV-tubulin (gold), basal cells with CK5 (magenta). White frame was magnified below. The green arrows indicate $ACE2^+/CC10^+$ cells, the yellow arrows indicate $ACE2^+/Muc5ac^+$ cells, the gold arrows indicate $ACE2^+$/beta-IV-tubulin$^+$ cells, the magenta arrows indicate $ACE2^+/CK5^+$ cells.
