# Supplementary material for: A pangolin-origin SARS-CoV-2-related coronavirus: infectivity, pathogenicity, and cross-protection by preexisting immunity
Source: Cell Discov. 2023 Jun 17;9:59. doi: 10.1038/s41421-023-00557-9 (PMC10276878; doi:10.1038/s41421-023-00557-9)
Supplement: Supplementary file 5 — Supplemental Fig S5 [file 41421_2023_557_MOESM5_ESM.pdf]

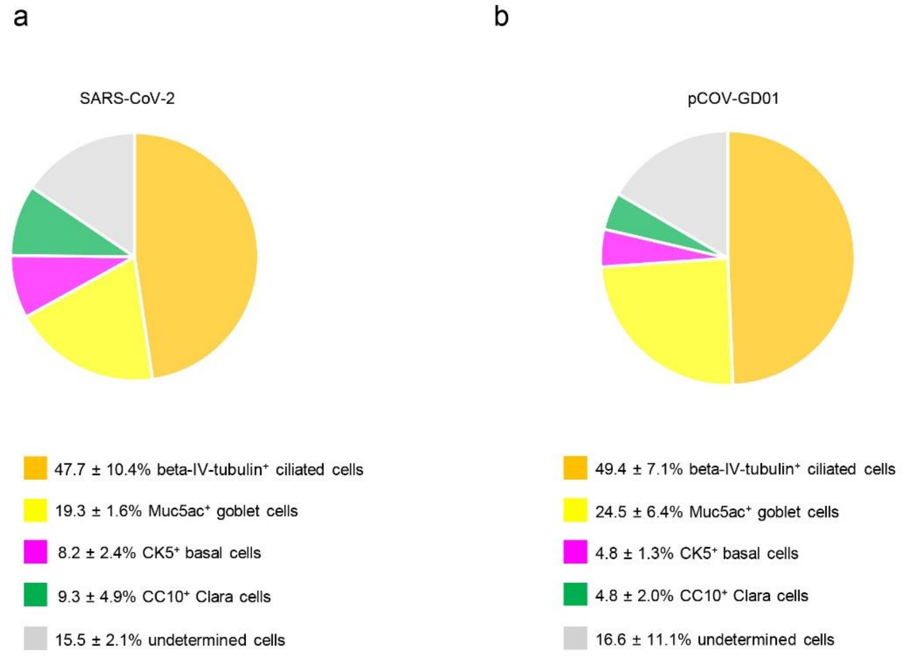

Supplementary Fig. S5 Percentage of each cell type with the SARS-CoV-2^+ or pCoV-$GD01^+$ population. a&b Statistical analysis of the percentage of each cell compartment within SARS-CoV-2^+ or pCoV-$GD01^+$ cells. The data are presented as means ± SD (n = 3).
