# Supplementary material for: A pangolin-origin SARS-CoV-2-related coronavirus: infectivity, pathogenicity, and cross-protection by preexisting immunity
Source: Cell Discov. 2023 Jun 17;9:59. doi: 10.1038/s41421-023-00557-9 (PMC10276878; doi:10.1038/s41421-023-00557-9)
Supplement: Supplementary file 6 — Supplemental Fig S6 [file 41421_2023_557_MOESM6_ESM.pdf]

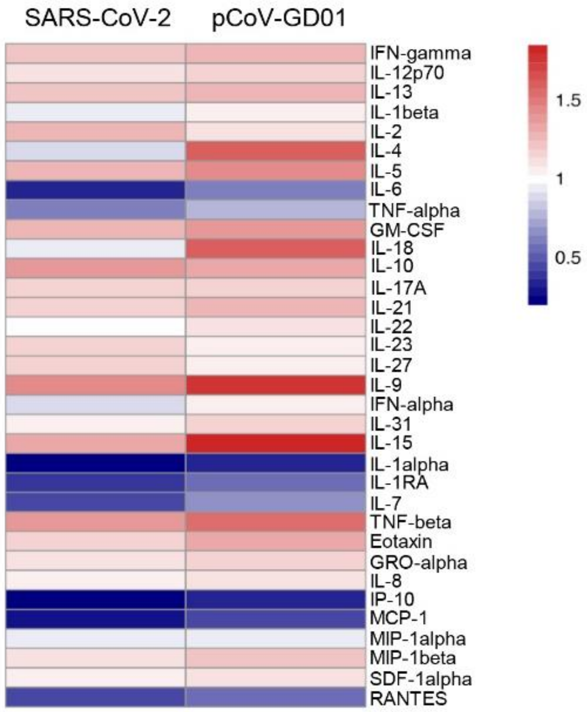

Supplementary Fig. S6 Cytokine production in topical secretions of pCoV-GD01, SARS-CoV-2 inoculated human airway epithelium organoids. Cytokine production in the topical secretions of human airway epithelium organoids at 24 hpi, the color represents fold change of detected cytokine concentration vs. that of MOCK samples.
