# Supplementary material for: A pangolin-origin SARS-CoV-2-related coronavirus: infectivity, pathogenicity, and cross-protection by preexisting immunity
Source: Cell Discov. 2023 Jun 17;9:59. doi: 10.1038/s41421-023-00557-9 (PMC10276878; doi:10.1038/s41421-023-00557-9)
Supplement: Supplementary file 7 — Supplemental Fig S7 [file 41421_2023_557_MOESM7_ESM.pdf]

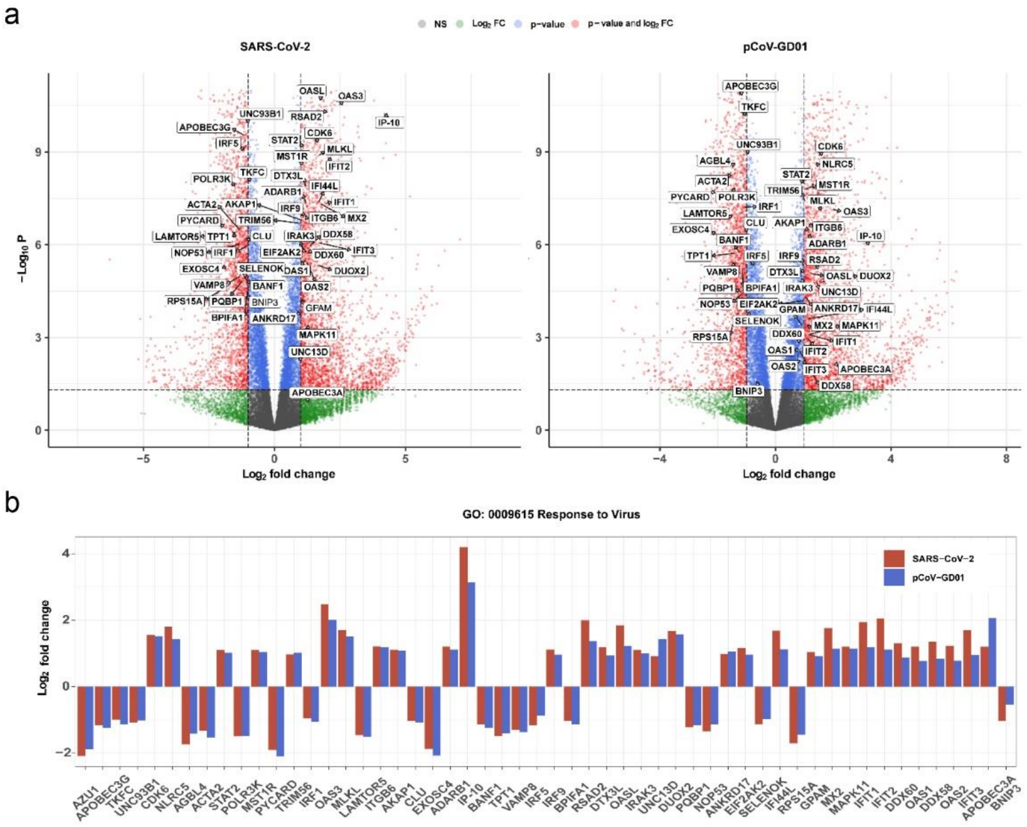

Supplementary Fig. S7 Differential expression genes in SARS-CoV-2- and pCoV-GD01-inoculated human airway epithelium organoids. a Volcano plots of differential expression genes in SARS-CoV-2- and pCoV-GD01- inoculated human airway epithelium organoids. The red point represent genes with their log2 fold change > 1 and p value <0.05. The labeled genes are the significant differential expression genes related with cellular response to virus. **b** Fold change of 55 overexpression or downregulation genes of GO:0009615 (response to virus） in SARS-CoV-2- and pCoV-GD01-inoculated human airway epithelium organoids.
