# Supplementary material for: A pangolin-origin SARS-CoV-2-related coronavirus: infectivity, pathogenicity, and cross-protection by preexisting immunity
Source: Cell Discov. 2023 Jun 17;9:59. doi: 10.1038/s41421-023-00557-9 (PMC10276878; doi:10.1038/s41421-023-00557-9)
Supplement: Supplementary file 8 — Supplemental Fig S8 [file 41421_2023_557_MOESM8_ESM.pdf]

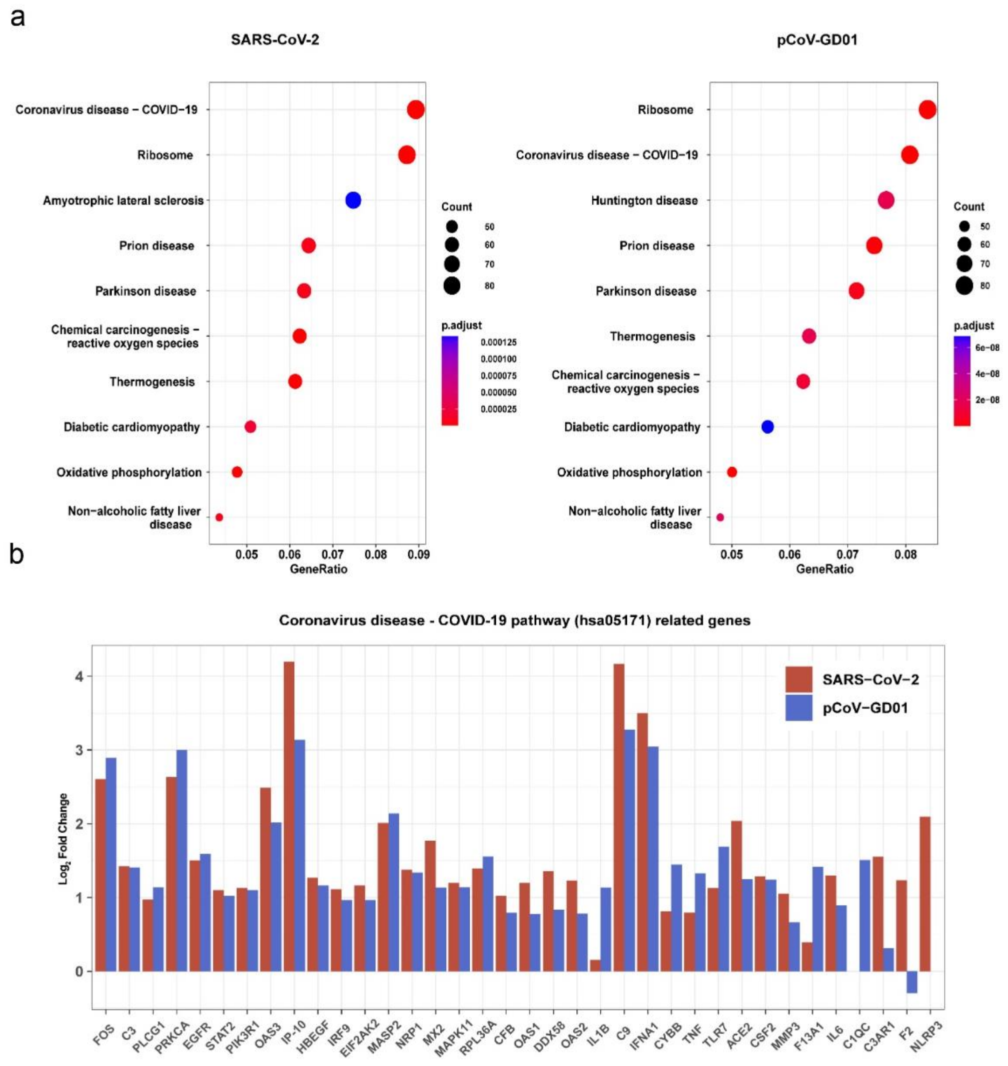

Supplementary Fig. S8 KEGG enrichment of differential expression genes in SARS-CoV-2- and pCoV-GD01-inoculated human airway epithelium organoids. a Dot plot of KEGG enrichment of differential expression genes in SARS-CoV-2- and pCoV-GD01- inoculated human airway epithelium organoids. The size of dots represents enriched genes count, while the color represents p-adjust value of each enriched category. **b** Fold change of the differential expression genes in KEGG pathway coronavirus disease—COVID-19.
