# Supplementary material for: A pangolin-origin SARS-CoV-2-related coronavirus: infectivity, pathogenicity, and cross-protection by preexisting immunity
Source: Cell Discov. 2023 Jun 17;9:59. doi: 10.1038/s41421-023-00557-9 (PMC10276878; doi:10.1038/s41421-023-00557-9)
Supplement: Supplementary file 9 — Supplemental Fig S9 [file 41421_2023_557_MOESM9_ESM.pdf]

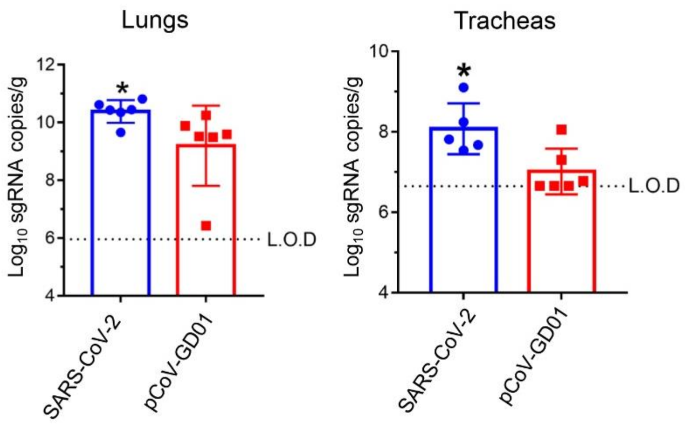

Supplementary Fig. S9 Viral sgRNA in the lungs and tracheas of SARS-CoV-2- or pCoV-GD01-inoculated hACE2 Mice. 8-week-old hACE2 mice (n = 6) at were inoculated with 6×10^3 PFU of pCoV-GD01 or SARS-CoV-2 through i.n. administration, and all mice were sacrificed at 72 hpi for lungs. Viral sgRNAs in the lungs and tracheas were determined by qRT–PCR. Data are shown as means ± SD. Student's t-test was performed for statistical analysis (*$p<0.05$). L.O.D, limit of detection.
