# Supplementary material for: A pangolin-origin SARS-CoV-2-related coronavirus: infectivity, pathogenicity, and cross-protection by preexisting immunity
Source: Cell Discov. 2023 Jun 17;9:59. doi: 10.1038/s41421-023-00557-9 (PMC10276878; doi:10.1038/s41421-023-00557-9)
Supplement: Supplementary file 10 — Supplemental Fig S10 [file 41421_2023_557_MOESM10_ESM.pdf]

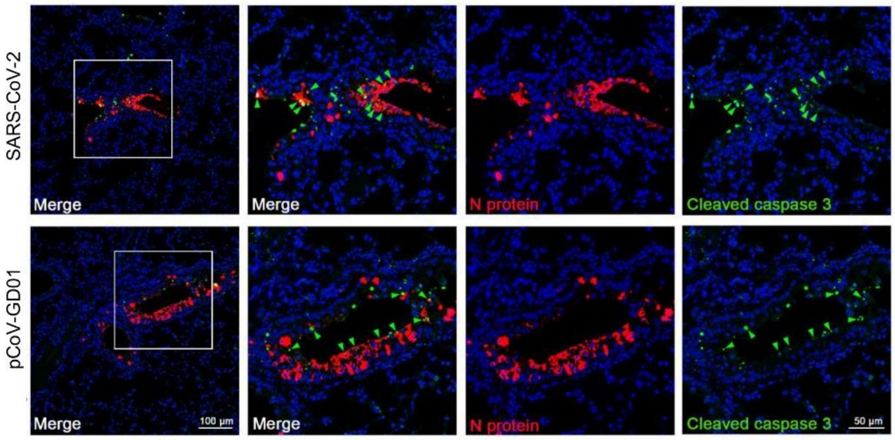

Supplementary Fig. S10 Pathological changes and inflammatory responses in pCoV-GD01-inoculated hACE2 Mice. Multiplex IF staining analysis for hACE2 mice paraffin lung sections with markers for cleaved-caspase3 (green) and N protein (red). White frame was magnified on the right. Green arrows indicated the N protein^{+}/C-Casp3^{+} cells.
