# Supplementary material for: A pangolin-origin SARS-CoV-2-related coronavirus: infectivity, pathogenicity, and cross-protection by preexisting immunity
Source: Cell Discov. 2023 Jun 17;9:59. doi: 10.1038/s41421-023-00557-9 (PMC10276878; doi:10.1038/s41421-023-00557-9)
Supplement: Supplementary file 11 — Supplemental Fig S11 [file 41421_2023_557_MOESM11_ESM.pdf]

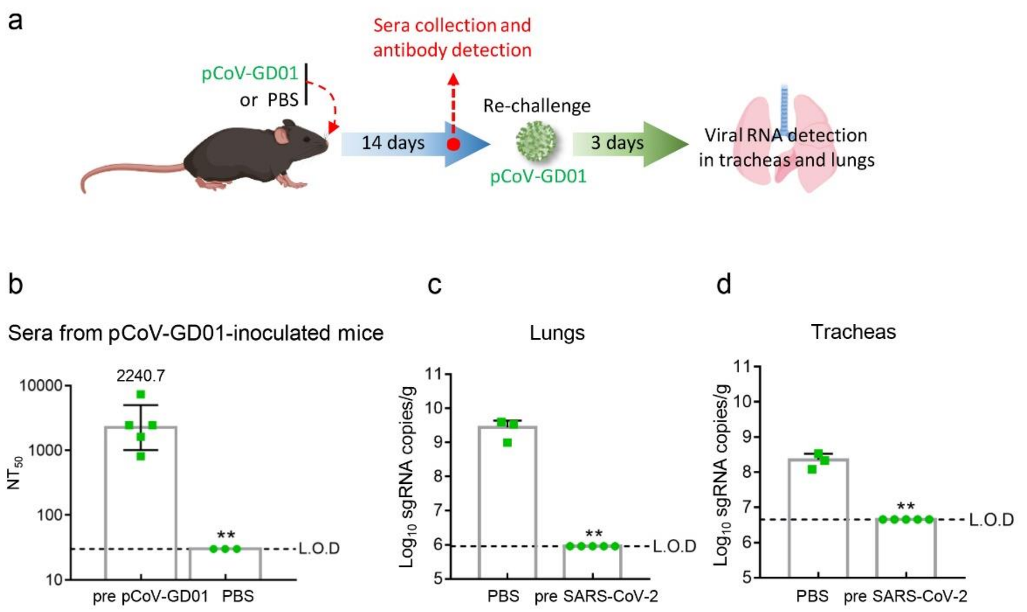

Supplementary Fig. S11 Pre-existing immunity of pCoV-GD01 protect against homologous challenge. a 8-week-old hACE2 mice were inoculated i.n. with 6×10^3 PFU pCoV-GD01 (n = 5) or PBS (n = 3), and 14 days later inoculated with an equivalent dose of pCoV-GD01. **b** The sera were collected at 14 dpi and subjected to neutralization assays. The NT_{50} of sera from were analyzed using pseudovirus for pCoV-GD01. Data are shown as geometric means ± SD. **c&d** Lungs and tracheas were collected 3 days post re-challenge. Viral sgRNA loads in the lungs and tracheas were determined by qRT–PCR. Data are shown as means ± SD. Student's t-test was performed for statistical analysis (**p<0.01). L.O.D, limit of detection.
