# Supplementary material for: A pangolin-origin SARS-CoV-2-related coronavirus: infectivity, pathogenicity, and cross-protection by preexisting immunity
Source: Cell Discov. 2023 Jun 17;9:59. doi: 10.1038/s41421-023-00557-9 (PMC10276878; doi:10.1038/s41421-023-00557-9)
Supplement: Supplementary file 12 — Supplemental Table S1 [file 41421_2023_557_MOESM12_ESM.pdf]

Supplementary Table S1 Primers used for qRT–PCR.

E_CoV_gRNA	Forward primer	ACAGGTACGTTAATAGTTAATAGCGT
	Reverse primer	ATATTGCAGCAGTACGCACACA
	Probe	ACACTAGCCATCCTTACTGCGCTTCG
E_CoV_sgRNA	Forward primer	CGATCTCTTGTAGATCTGTTCTC
	Reverse primer	ATATTGCAGCAGTACGCACACA
	Probe	ACACTAGCCATCCTTACTGCGCTTCG
