# Supplementary material for: A pangolin-origin SARS-CoV-2-related coronavirus: infectivity, pathogenicity, and cross-protection by preexisting immunity
Source: Cell Discov. 2023 Jun 17;9:59. doi: 10.1038/s41421-023-00557-9 (PMC10276878; doi:10.1038/s41421-023-00557-9)
Supplement: Supplementary file 13 — Supplemental Table S2 [file 41421_2023_557_MOESM13_ESM.pdf]

**Supplementary Table S2 Sequence information of CoVs Supplementary Fig. S1.**

| CoV strains | Accession ID in GISAID |
|---|---|
| pCoV-GD01 | EPI_ISL_410721 |
| pCoV-GD/MP789 | EPI_ISL_412860 |
| pCoV-GX/P1E | EPI_ISL_410539 |
| pCoV-GX/P2V | EPI_ISL_410542 |
| pCoV-GX/P4L | EPI_ISL_410542 |
| pCoV-GX/P5E | EPI_ISL_410541 |
| pCoV-GX/P5L | EPI_ISL_410540 |
| bCoV-Laos/BANAL-20-52 | EPI_ISL_4302644 |
| bCoV-Laos/BANAL-20-236 | EPI_ISL_4302647 |
