# Supplementary material for: A pangolin-origin SARS-CoV-2-related coronavirus: infectivity, pathogenicity, and cross-protection by preexisting immunity
Source: Cell Discov. 2023 Jun 17;9:59. doi: 10.1038/s41421-023-00557-9 (PMC10276878; doi:10.1038/s41421-023-00557-9)
Supplement: Supplementary file 14 — Supplemental Table S3 [file 41421_2023_557_MOESM14_ESM.pdf]

Supplementary Table S3 Details of Convalescent sera.

Patient NO.	Sex	Age	Diagnosis
29	male	75	severe
34	male	64	mild
7	male	32	mild
2	Female	29	mild
4	male	35	mild
5	male	68	mild
9	male	37	mild
14	male	37	mild
15	male	37	mild
17	Female	53	mild
18	male	44	mild
20	male	53	mild
21	male	66	mild
23	male	32	mild
25	male	40	severe
27	Female	53	mild
30	Female	53	mild
31	male	64	mild
33	male	36	severe
